# The effect of XC-running race Lidingöloppet on determinants of performance

**DOI:** 10.3389/fphys.2025.1647810

**Published:** 2025-10-07

**Authors:** Elias Rapp, Francesco Laterza, Vincenzo Manzi, Ferdinand Von Walden, Daniele A. Cardinale

**Affiliations:** ^1^ Department of Physiology, Nutrition and Biomechanics, The Swedish School of Sport and Health Sciences GIH, Stockholm, Sweden; ^2^ Department of Education and Sport Sciences, Pegaso Open University, Naples, Italy; ^3^ Department of Neurosciences, Biomedicine and Movement Sciences, University of Verona, Verona, Italy; ^4^ Department of Women’s and Children’s Health, Karolinska Institutet, Stockholm, Sweden; ^5^ Department of Physiology and Pharmacology, Karolinska Institutet, Stockholm, Sweden; ^6^ The Swedish Sports Confederation (Riksidrottsförbundet), Stockholm, Sweden

**Keywords:** running economy, running, off-road running, physiological resilience, VO_2_max

## Abstract

**Aim:**

This study aimed to investigate the determinants of running performance in a cross-country running race and examine whether running economy and biomechanics are affected. Moreover, we analyzed whether the magnitude of change in running economy (RE) is related to changes in biomechanics, performance, and fitness measures.

**Method:**

Thirteen runners (12 male and 1 female), with an average 10 km personal best time of 36:46 ± 3:17 (min:s), participated in the 30 km cross-country race, Lidingöloppet. Assessments of submaximal and maximal running physiology, biomechanics, and anthropometry were conducted before and immediately after the race. A multiple linear regression model was applied to explain performance variance. Pearson’s correlation analyses examined the relationships between performance and pre-test variables, and between changes in running economy and both pre-test fitness measures and changes in biomechanics. Paired Student’s t-tests were used to compare pre- and post-race values.

**Results:**

Performance was best explained using a model including oxygen uptake at lactate threshold (LT), fat utilization, and allometrically scaled running economy (*R* ^2^ = 0.918, adjusted *R* ^2^ = 0.887, F = 29.7, *p* < 0.01). Race performance also correlated with maximal oxygen uptake (VO_2_max, r = −0.776, *p* = 0.003), fat mass (r = 0.646, *p* = 0.032), and velocity at VO_2_max (vVO_2_max, r = −0.853, *p* < 0.01). The oxygen cost of running increased (201.8 ± 14 vs. 208.4 ± 9.3 mL kg^−1^·km^−1^; *p* = 0.041), whereas respiratory exchange ratio (0.91 ± 0.04 vs. 0.85 ± 0.05; *p* < 0.01) and body mass (69.2 ± 7.5 vs. 67.6 ± 7.7 kg; *p* < 0.01) decreased post-race. Energetic cost of running (0.997 ± 0.076 vs. 1.015 ± 0.052 kcal kg^−1^·km^−1^; *p* = 0.192) and all biomechanical measurements, including cadence, contact time, overstride, vertical displacement, and vertical force, were unaffected by the race. The magnitude of change in running economy was related only to pre-test running economy (r = −0.749; *p* = 0.003) but not to performance (r = −0.440; *p* = 0.132), other pre-test fitness measures, or any changes in biomechanics.

**Conclusion:**

The best performance prediction model included oxygen uptake at estimated lactate threshold, fat utilization during submaximal running, and allometrically scaled running economy. Oxygen cost of running increased post-race, likely due to increased fat oxidation, despite decreased body mass. No changes in biomechanics were observed, and changes in running economy could not be explained by changes in biomechanics. Aerobic fitness, anthropometry, and performance were not associated with changes in running economy. Given the small and relatively homogeneous sample, findings should be considered exploratory, although they suggest that practitioners may benefit from targeting fat oxidation, oxygen uptake at the estimated lactate threshold, and running economy in training.

## Introduction

Trail and off-road running races are typically held on undulating terrain with uneven surfaces, which may affect running mechanics and, consequently, physiological responses (Ehrström et al., 2018). Although many studies on long-distance running have focused on flat-ground performance—identifying maximum oxygen uptake (VO_2_max), the fraction of VO_2_max sustained during performance (fractional utilization, related to lactate and ventilatory thresholds), and running economy (RE) as key determinants (Bassett and Howley, 2000; Joyner and Coyle, 2008; Mclaughlin et al., 2010)—comparatively few investigations have explored performance determinants in off-road conditions, especially in races that are not extremely demanding in terms of elevation change, terrain, or duration.

The classical model of running performance appears less predictive for trail events conducted in hilly terrain (de Waal et al., 2021). In these settings, RE and variables related to fractional utilization generally do not predict performance as strongly as VO_2_max. For example, one study found that performance in a 27-km race (with a 1,400-m elevation gain) was best explained using VO_2_max, local muscle endurance (measured as a fatigue index in the knee extensor muscle through a test involving 40 consecutive maximal concentric contractions), and RE on a 10% slope (Ehrström et al., 2018). In another study, VO_2_max and the percentage of fat mass were the strongest predictors for performance in a 27-km event with a 1,700-m elevation gain (Alvero-Cruz et al., 2019). Similarly, in 40- and 55-km races starting at approximately 1,000 m altitude and involving 2,300–3,500 m of elevation gain, VO_2_max and fat substrate utilization at 10 km h^−1^ emerged as the strongest predictors (Pastor et al., 2022). VO_2_max and the peak running velocity achieved in the incremental test were also associated with performance, while RE was not (Coates et al., 2021). In a shorter-duration, sea-level 7-km trial with a 486-m elevation gain, performance was associated with VO_2_max, vertical uphill speed, lean mass, and body fat mass percentage (Björklund et al., 2019). Although the studies differ slightly in terms of the most relevant variables, they consistently highlight that RE does not predict performance, while VO_2_max remains a key parameter (Ehrström et al., 2018; Alvero-Cruz et al., 2019; Pastor et al., 2022; Coates et al., 2021). Only one study suggested that lactate threshold (LT) might also be important in XC running over 31 km, with a 550-m elevation gain (Scheer et al., 2019).

It should be noted that these studies typically assess physiological determinants such as RE in a non-fatigued state. Maintaining RE over time during a prolonged effort appears to be crucial for optimal performance, with fatigue resilience emerging as an additional determinant of endurance (Jones et al., 2021; Brueckner et al., 1991). Moreover, the ability to sustain a critical speed throughout long-distance running can differentiate athletes with similar pre-competition capabilities (Jones, 2024), emphasizing the need to evaluate performance under fatigue. However, findings regarding how RE responds to fatigue remain inconsistent across studies. Increases in oxygen uptake or energy cost at a given speed have been reported following short flat treadmill trials (approximately 12.5 min, 60 min, or even 24 h in length) (Candau et al., 1998; Hunter and Smith, 2007; Gimenez et al., 2013), flat road marathons (Brueckner et al., 1991; Kyröläinen et al., 2000; Nicol et al., 2007), and submaximal flat track running (Xu and Montgomery, 1995). Trail running also shows mixed results, with some studies reporting worsened RE after 40- and 55-km races with 2,300–3,500 m of elevation gain (Sabater Pastor et al., 2021) and after a 43-km uphill race with 3,000 m of elevation gain (Lazzer et al., 2015), while other investigations have noted decreased oxygen and energy costs after an ultramarathon of 330 km or no significant changes in several races longer than 65 km (Vernillo et al., 2017). These findings suggest that both the duration and the relative intensity of the race may affect the cost of running, with higher intensities leading to a larger increase in cost and longer races showing a smaller increase (Xu and Montgomery, 1995; Sabater Pastor et al., 2021). Notably, one study found a positive correlation between race speed in trail races of 40 km, 55 km, and over 100 km and the degree of change in RE. This result contradicts the notion that more proficient athletes exhibit less deterioration in RE (Sabater Pastor et al., 2021). Additionally, although individual variation in RE change appears to be influenced by the mechanical power of the lower limb—where higher power mitigates deterioration (Lazzer et al., 2015)—the role of aerobic fitness and potential biomechanical adjustments remains unclear as some studies report no relationship between changes in biomechanics and RE (Hunter and Smith, 2007; Kyröläinen et al., 2000; Nicol et al., 2007), whereas others indicate a connection (Lazzer et al., 2015; Vernillo et al., 2017).

Therefore, the purpose of this study was to examine the determinants of performance in a less “extreme” XC running race while investigating the effects of such a race on RE and running biomechanics. A further aim was to analyze the relationship between biomechanical changes and alterations in RE and assess potential links between the degree of RE change, race performance, and physiological fitness measures. To this end, the study focuses on the classical XC running race Lidingöloppet—a 30-km race with a 550-m elevation gain, run on gravel roads and grass on Lidingö island (Stockholm, Sweden) at sea level.

## Methodology

### Experimental overview

**FIGURE 1:**
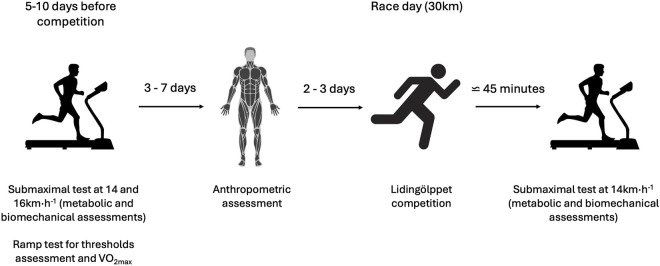
Timeline and flowchart of the experimental protocol.

The research was conducted using a single-group repeated-measures, pre–post-test design, in which each participant was assessed before and after the intervention. All participants completed three test sessions: two test sessions within 2 weeks, in a rested state, prior to the Lidingöloppet race, and one post-test immediately after the race. The first test session, performed 5–10 days prior to Lidingöloppet, consisted of a treadmill running test to measure submaximal and maximal physiological and biomechanical parameters. The second test session consisted of an anthropometric assessment of body composition and was performed 3–7 days following the first test. Post-tests were conducted immediately after the race in a field-laboratory constructed at the finish line of the race. The Swedish Ethical Review Authority approved the study (2022-04035-01), and participants provided verbal and written consent after being informed of any potential risks associated with the experiments prior to participation. An overview of the experimental design is provided in Figure 1.

### Participants

Thirteen runners (twelve male and one female, average age 35 ± 4 years, 10 km PB = 36:46 ± 03:17 min:ss) were recruited for the study through digital advertising on social media, sports associations, and sports clubs. Participants were included if they were healthy individuals between 18 and 40 years old, willing to participate in the 30 km cross-country race “Lidingöloppet,” and able to verify their 10 km personal best. Individuals were excluded if they had any current or recent injury (within the past 6 months), were smokers, or had a 10 km personal best slower than 40 min, as verified through official race results or GPS files.

### Data collection

#### Pre-tests

Participants arrived at the laboratory in a rested state, where their body mass and height were measured. Participants were instructed to follow the following standardization guidelines before testing: avoid any high-exertion training for 3 days prior to the test, consume the same pre-test meal for each session, refrain from eating during the 2 h preceding the test, and avoid caffeine for at least 3 h beforehand. The same pair of running shoes—relatively new and minimally worn—were to be used for both pre- and post-tests to minimize their impact on RE (Black et al., 2022). After these preparations, participants completed a treadmill running test to assess running biomechanics, the ventilatory marker of LT (Keir et al., 2022), i.e., θLT and respiratory compensation point (RCP), along with maximal physiological responses. The treadmill test started with a 5-min warm-up at a self-selected speed corresponding to a 10–12 rating of perceived exertion (RPE, on the Borg RPE scale) (Borg, 1990), followed by 3 min at a self-selected speed corresponding to 13–15 RPE, and finished with 3 min of rest. Then, a submaximal part began, consisting of two stages of 5 min running at 14 km h^−1^ and 16 km h^−1^ for measurements of cardiopulmonary variables using a metabolic-cart system equipped with a mixing chamber (COSMED, Quark CPET, Italy). During the submaximal phase, biomechanical variables (clarified in a later section) were also measured using a markerless motion capture system (MotionMetrix, 3D running gait, Sweden), a Stryd sensor (Stryd Powermeter; Stryd, Inc., Boulder, CO), and a built-in force transducer in the treadmill. Each 5-min section was followed by a 1-min break for the collection of a capillary blood sample for lactate measurement (H-la, EKF Diagnostics, Biosen C-line, United Kingdom) and assessing RPE (Borg, 1990). The test was terminated if the respiratory exchange ratio (RER) during the 5-min stage exceeded 1.0. Six athletes failed to complete the 16 km h^−1^ stage with an RER below 1.0, whereas all athletes completed the 14 km h^−1^ stage with an RER below 1.0 (Table 1). Following the two 5-min submaximal runs, there was a break consisting of 5 min of rest, followed by a maximal incremental exercise test. The maximal incremental exercise test started at 10 km h^−1^, and the speed was increased by 0.4 km h^−1^ every 30 s until task failure. RPE was collected shortly after task failure, and blood lactate was measured 1 and 3 min after task failure.

**TABLE 1:** Pre-test anthropometric and physiological data from both submaximal and maximal tests and results of correlation analysis between each variable and race time.

	Pre-test variable	Mean ± SD	Pearson’s r	*r* ^2^	*p*-value
Anthropometric characteristic and their relationship to race performance	Height (cm)	175.2 ± 7.1	0.213	0.05	0.486
	Body mass (kg)	69.2 ± 7.5	0.378	0.14	0.203
	Body fat %	16.1 ± 3.1	0.510	0.26	0.109
	Fat mass (kg)	11.0 ± 2.2	0.646*	0.42	0.032
	Lean mass %	80.0 ± 3.0	−0.483	0.23	0.132
	Lean mass (kg)	54.88 ± 5.96	0.072	0.01	0.833
Pre-test physiological characteristics from submaximal running at 14 km h^−1^ and correlation with performance	VO_2_ (L·min^−1^)	3.27 ± 0.45	0.588*	0.35	0.035
	VO_2_ (mL·min^−1^·kg^-1^)	47.1 ± 45.7	0.554*	0.31	0.05
	RER	0.91 ± 0.04	0.660*	0.44	0.014
	Fat%	28.8 ± 13.0	−0.659*	0.43	0.014
	Carb%	71.2 ± 13.0	0.659*	0.43	0.014
	RE_ox_ (mL·kg^−1^·km^−1^)	201.8 ± 14	0.550	0.30	0.051
	RE_allometric_ (mL/kg^0.75^/km)	581.8 ± 46.3	0.614*	0.38	0.026
	RE_ec_ (kcal·kg^−1^·km^−1^)	0.997 ± 0.076	0.591*	0.35	0.033
Pre-test physiological characteristics from the maximal incremental test and correlation with performance	VO_2_max (mL·min^−1^·kg^−1^)	62.24 ± 6.17	−0.776*	0.60	0.003
	vVO_2_max (km·h^−1^)	18.9 ± 2.1	−0.853*	0.73	<0.001
	vPeak (km·h^−1^)	20.1 ± 2.0	−0.829*	0.69	<0.001
	vθLT (km·h^−1^)	14.7 ± 1.8	−0.842*	0.71	<0.001
	VO_2_ at θLT (mL·min^−1^·kg^−1^)	50.10 ± 5.96	−0.743*	0.55	0.006
	vRCP (km·h^−1^)	16.6 ± 2.2	−0.812*	0.66	0.002
	VO_2_ at RCP (mL·min^−1^·kg^-1^)	56.18 ± 6.13	−0.680*	0.46	0.021

*p ≤ 0.05; RER, respiratory exchange ratio; RE_ox_, running economy oxygen cost; RE_allometric_, running economy allometrically scaled oxygen cost; RE_ec_, running economy energy cost; θLT, estimated lactate threshold; RCP, respiratory compensation point; vPeak, peak running velocity achieved in the incremental test.

Before each treadmill test, the metabolic-cart system, the blood lactate measurement device, and the markerless motion capture system were calibrated according to the manufacturer’s guidelines.

At the second test session, body composition was measured via dual-energy x-ray absorptiometry (DEXA; Horizon Hologic, United States), including body fat %, body fat (kg), lean mass %, and lean mass (kg). The participants were instructed to fast for at least 5 h before the test and to refrain from alcohol and intensive training likely to cause excessive sweating on the preceding evening.

### The race Lidingöloppet

Following the baseline assessment, the participants completed the XC running race Lidingöloppet—a 30-km race with a 550-m elevation gain, run on gravel roads and grass on Lidingö island (Stockholm, Sweden) at sea level. During the race, the runners aimed to perform maximally. Performance during the race was measured as the time to completion from the race start, based on the official chip time. The temperature during the race was ∼14 °C, with no rainfall.

### Post-race test

At the finish line of the race, an in-field laboratory was set up inside a tent to ensure stable environmental conditions. Following race completion, the participants immediately entered the in-field laboratory for the measurement of body mass. Thereafter, the athletes completed 5-min of submaximal running at 14 km h^−1^. For the measurement of physiological and biomechanical variables, the same equipment and standardization procedures as in the pre-test were used. Only one athlete could be tested at a time; therefore, participants were tested in the order they finished the race. This resulted in an average waiting time of 45:15 ± 24:28 (min:ss) following race completion.

### Data analysis

#### Physiological parameters

Submaximal values of RER, VO_2_ (mL·min^−1^·kg^−1^ and L·min^−1^), VE (L·min^−1^), and breathing frequency (L/min) were collected during the final minute of the 5-min stage at 14 km h^−1^, both pre- and post-test. Due to the low number of participants finishing the 16 km h^−1^ stage, no analysis was performed on these data. Thus, all presented analyses refer to the 14 km h^−1^ condition. To ensure valid measurements of submaximal variables during the 14 km h^−1^ runs, data from each participant were analyzed for steady state, i.e., an increase in VO_2_ of less than 100 mL min^−1^·kg^−1^ during the last minute of exercise (Fletcher et al., 2009). Based on metabolic gas exchange data, RE was expressed as either oxygen cost (RE_*ox*_) or energy cost in kilocalories (RE*ec*) per kilogram of body mass per kilometer at a given speed (RE_*ox*_, mL·kg^−1^·km^−1^ (Foster and Lucia, 2007) using the following equation:

$${\mathrm{RE}}_{\mathrm{ox}}={\mathrm{VO}}_{2}\;/\;m\times \min^{-1}\times 1000.$$

RE, expressed as energy cost [RE_ec_, kcal·kg^−1^·km^−1^ (Fletcher et al., 2009)], was calculated from VO_2_ (L·min^−1^), RER, and the caloric equivalent of VO_2_ (Lusk, 1924), body mass (kg), and running speed (m/min) using the following equation:

$${\mathrm{RE}}_{\mathrm{ec}}=\frac{{\mathrm{VO}}_{2}\times \mathrm{caloric}\;\mathrm{equivalent}}{m\times \min^{-1}}\times \frac{1000}{\mathrm{body}\;\mathrm{mass}}.$$

RE, expressed as allometrically scaled oxygen cost [RE_allometric_, mL·kg^−0.75^·km^−1^ (Svedenhag and Sjödin, 1994)], was calculated from running speed (m/min), VO_2_ (mL·min^−1^·kg^−1^), and allometrically scaled body mass using the following equation:

$${\mathrm{RE}}_{\mathrm{allometric}}={\mathrm{VO}}_{2}\;\left(\mathrm{mL}/{\mathrm{kg}}^{0.75}/\min\right)\;/\;m\times \min^{-1}\times 1000.$$

The specific scaling of body mass was arbitrarily chosen based on the research by Svedenhag and Sjödin (1994).

Furthermore, the energy cost from work of breathing (wb, kcal·kg^−1^·km^−1^) was calculated from VE (L·min^−1^), body mass (kg), and running speed (m/min) using the following equation adapted from Coast et al. (1993):

$$\mathrm{wb}=‐0.251+0.0382\times \mathrm{VE}+0.00176\;\cdot \;{\mathrm{VE}}^{2}\;/\;4184\;/\mathrm{kg}/\;m\times \min^{-1}\times 1000.$$

Moreover, the energetic cost of running (RE_ec-wb_) without wb was calculated by subtracting wb from RE_ec_, RE_ec_–wb.

During the maximal incremental test, VO_2_max was determined as the highest 30-s rolling average of VO_2_ [as previously applied, for example, by Jones et al. (2021)] for (mL·min^−1^·kg^−1^ and L·min^−1^). VO_2_max was defined as a plateau in VO_2_, despite increased workload. The plateau was visually determined. Moreover, the first speed to elicit VO_2_max was determined as vVO_2_max. VO_2_ and speed at θLT and RCP were determined during the maximal incremental test through analyses of VE, VCO_2,_ ventilatory equivalent of VO_2_ (VE/VO_2_), and VCO_2_ (VE/VCO_2_), along with end-tidal pressure of O_2_ (PETO_2_) and CO_2_ (PETCO_2_), as described by Keir et al. (2022).

#### Biomechanical parameters

During the pre-and post-test, the mean values of the following variables, collected using MotionMetrix, were analyzed during steady-state running between 3:30 and 3:50 of the 5-min stages: cadence (spm), ground contact time (ms), overstride (cm), vertical displacement (cm), and vertical force (bw). No side differences were analyzed; rather, the mean between both sides was used. These variables were chosen based on the reliability and validity of the system, which are discussed in the next section.

During the submaximal test at 14 km h^−1^ on the pre-test day, each runner wore a Stryd sensor (Stryd Powermeter; Stryd, Inc., Boulder, CO), securely attached to the shoe, according to the manufacturer’s recommendations. The sensor streams data at 1 Hz and records variables including running power output, ground contact time, vertical oscillation, leg spring stiffness, cadence, and step length. According to Stryd’s technical specifications, no calibration was required beyond entering the runner’s basic anthropometrics, and the device’s reported measurement error is approximately 3%. Data extraction was accomplished following the manufacturer’s guidelines.

### Statistical analyses

Data were tested for normality using the Shapiro–Wilk test. For analysis of variables related to performance, a hierarchical multiple linear regression model was used to explain variance in performance. Independent variables were added in order of their correlation to performance. To limit collinearity in the model, the variance inflation factor and tolerance of the regression model were calculated, with an upper limit of 2.0 for the variance inflation factor and a lower limit of 0.5 for tolerance. The regression model resulting in the highest *R* ^2^ without collinearity between variables will be presented. Correlation analyses were performed between physiological variables, Stryd biomechanical variables (power output, ground contact time, vertical oscillation, leg spring stiffness, cadence, and step length), and performance using Pearson’s r. Differences between pre–post tests for physiological and biomechanical variables at 14 km h^−1^ were analyzed using paired sample t-tests for normally distributed variables and Wilcoxon rank for skewed variables. To study the relationship between changes in biomechanical variables and changes in RE, computed Δ variables (post−pre) for RE and biomechanics were analyzed using Pearson’s r correlation. If skewed, Spearman’s rho was used. To further investigate changes in RE, correlation analyses using Pearson’s r were performed between ΔRE and pre-test measures of VO_2_max (mL·min^-1^·kg^-1^), VO_2_ at θLT, RE_ox_, RER, and race time (s). Furthermore, correlations between ΔRE and ΔRER, ΔVE (L·min^-1^), Δwb (kcal·kg^-1^·km^-1^), and Δbody mass (kg) were analyzed. Statistical significance was set at *p* ≤ 0.05. Data are presented as the mean ± SD, if not stated otherwise. All analyses were performed using Jamovi (2.3.21, The Jamovi Project, 2022).

## Results

### Race performance and participant characteristics

The participants ran the race with an average speed of 13.8 ± 1.7 km h^−1^, resulting in an average finishing time of 02:12:20 ± 00:14:48 (hr:min:ss), 35% ± 15% slower than the winning time and with a race placement of 398 ± 342.

Pre-test anthropometric and physiological data from both submaximal and maximal tests, along with the results of the correlation analysis between each variable and race time, are presented in Table 1. The only anthropometric variable related to performance was body fat mass (kg) (r = 0.646; *p* = 0.032). All other anthropometric variables were not related to race performance. All the pre-test physiological variables considered were correlated with the race time under both conditions (submaximal and maximal), except for RE_ox_. No significant correlation was found between the race time and power output, ground contact time, vertical oscillation, leg spring stiffness, cadence, or step length measured using the Stryd device.

### Race performance regression models

The multiple regression model, with the race time as a dependent variable resulting in the highest *R* ^2^, is shown in Table 2. After controlling for collinearity, the best regression model included the independent variables Fat % at 14 km h^-1^ and VO_2_ at θLT and RE_allometric_. This model explained 91.8% of the variance in the race time (*R* ^2^ = 0.918, adjusted *R* ^2^ = 0.887, F = 29.7, and *p* < 0.001). The results of the classical endurance performance model, including VO_2_max, RE_ox_, and VO_2_ at θLT, are presented in Table 3. Collinearity between variables existed, and *R* ^2^ did not reach above 0.918 (*R* ^2^ = 0.834, adjusted *R* ^2^ = 0.772, F = 13.4, and *p* = 0.002). Although the studied group was fairly homogeneous, with most ages clustering between the early and late 30s and a CV of only ∼11%, additional analyses including age as a covariate showed that age was not a significant predictor of the race time (*p* = 0.566) and did not improve model fit (adjusted *R* ^2^ = 0.877 vs. 0.887 without age).

**TABLE 2:** Multiple regression analysis with race time (s) as a dependent variable.

Independent variable	Estimate (SE)	p	VIF	Tolerance
Intercept	10835.95 (1859.49)	<0.001	-	-
Fat% at 14 km h^−1^	−27.79 (8.89)	0.014	1.64	0.609
VO_2_ at θLT	−101.93 (15.67)	<0.001	1.02	0.984
RE_allometric_	5.2 (2.54)	0.075	1.64	0.609

θLT, estimated lactate threshold; RE_allometric_, running economy allometrically scaled oxygen cost; VIF, variance inflation factor.

**TABLE 3:** Classical model of endurance performance with race time (s) as a dependent variable.

Independent variable	Estimate (SE)	p	VIF	Tolerance
VO_2_max	−101.85 (62.76)	0.143	8.69	0.115
VO_2_ at θLT	−8.26 (65.01)	0.902	8.69	0.115
RE_ox_	30.49 (9.15)	0.010	1.01	0.992

θLT, estimated lactate threshold; RE_ox_, running economy oxygen cost; VIF, variance inflation factor.

### Post-race changes

**FIGURE 2:**
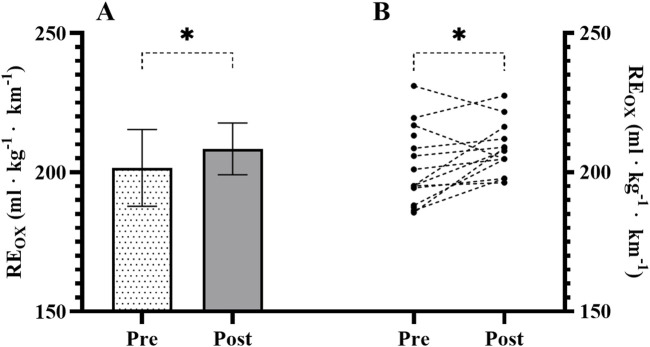
**(A)** Mean ± SD of running economy oxygen cost (RE_ox_) during the pre- and post-test at 14 kmh^−1^. **(B)** Individual values of RE_ox_ at 14 kmh^−1^ during the pre- and post-test. *Significant difference (*p* ≤ 0.05) between the pre- and post-test.

**FIGURE 3:**
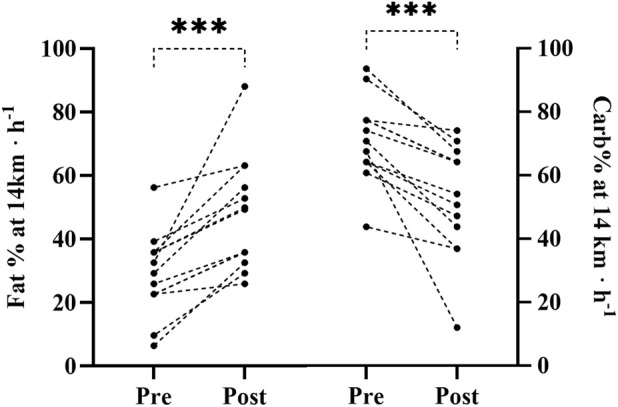
(Left panel) Participants’ individual fat utilization (fat%) at 14 kmh^−1^ in the pre-and post-test. (Right panel) Participants’ individual carbohydrate utilization (carb%) at 14 kmh^−1^ in the pre- and post-test. ***Significant difference (*p* ≤ 0.001) between the pre- and post-test for group mean.

All participants were tested after finishing the race. Physiological variables measured in both pre- and post-tests are presented in Table 4. Post-race VO_2_ relative to body mass (mL· min^−1^·kg^−1^) increased significantly by 3.4%, whereas absolute VO_2_ (L·min^−1^, −0.73%) and VE (4.55%) showed no significant changes. The race also negatively affected RE_ox_, which increased by 3.4% from pre- to post-race (Figure 2). However, when running economy was allometrically scaled (RE_allometric_), it did not differ significantly (2.5%). Furthermore, RE_ec_ also showed no significant difference between pre- and post-tests (2.1%). Substrate utilization (Figure 3) changed significantly post-race with decreased RER (−6.18%), increased Fat% (62.22%), and decreased Carb% (−25.32%).

**TABLE 4:** Pre- and post-test physiological data from submaximal running at 14 kmh^-1^ and paired Student’s t-test results.

	Pre-test mean ± SD	Post-test mean ± SD	Δ (post−pre) Mean 95% CI [LL, UL]	t, df	Effect size (Cohen’s d)	*p*-value
VO_2_ (L·min^−1^)	3.27 ± 0.45	3.29 ± 0.43	0.02 [−0.08, 0.13]	0.482, 12	0.134	0.639
VO_2_ (mL·min^−1^·kg^−1^)	47.1 ± 3.3	48.6 ± 2.2	1.5 [0.07, 3.01]	2.284, 12	0.634*	0.041
VE (L·min^−1^)	91.33 ± 17.83	95.9 ± 14.98	4.57 [−0.54, 9.67]	1.949, 12	0.541	0.075
Breathing frequency (L·min^−1^)	42.63 ± 6.63	50.5 ± 5.52	7.42 [4.74, 10.11]	6.024, 12	1.671*	<0.01
RER	0.91 ± 0.04	0.85 ± 0.05	−0.06 [−0.08, −0.03]	−5.083, 12	−1.410*	<0.01
Fat%	28.8 ± 13.0	47.5 ± 17.5	18.71 [10.64, 26.78]	5.050, 12	1.4*	<0.01
Carb%	71.2 ± 13.0	52.8 ± 17.5	−18.4 [−26.57, −10.23]	−4.908, 12	−1.361*	<0.01
H-la (mmol·L^−1^)	2.2 ± 0.89	2.82 ± 1.0	0.63 [0.11, 1.14]	2.659, 12	0.737*	0.021
HR (bpm)	156 ± 17	166 ± 11	6 ± [−6, 17]	1.139, 8	0.380	0.288
WB (kcal·kg^−1^·km^−1^)	0.075 ± 0.022	0.083 ± 0.016	0.008 [−0.00, 0.02]	2.382, 12	0.661*	0.035*
RE_ox_ (mL·kg^−1^·km^−1^)	201.8 ± 14	208.4 ± 9.3	6.6 [0.31, 12.99]	2.284, 12	0.633*	0.041
RE_allometric_ (mL/kg^0.75^/km)	581.8 ± 46.3	597.7 ± 35.9	16.0 [−1.56, 33.47]	1.985, 12	0.550	0.071
RE_ec_ (kcal·kg^−1^·km^−1^)	0.997 ± 0.076	1.015 ± 0.052	0.019 [−0.01, 0.05]	1.369, 12	0.380	0.196
RE_ec-wb_ (kcal·kg^−1^·km^−1^)	0.922 ± 0.059	0.933 ± 0.043	0.011 [−0.01, 0.06]	1.389, 12	0.385	0.190
Body mass (kg)	69.2 ± 7.5	67.6 ± 7.7	−1.6 [−2.13, −1.13]	−7.127, 12	−1.977*	<0.01

*p ≤ 0.05; 95% CI, 95% confidence interval; LL, lower limit; UL, upper limit; df, degrees of freedom; VE, ventilation; RER, respiratory exchange ratio; H-la, hemo lactate; HR, heart rate; WB, work of breathing; RE_ox_, running economy oxygen cost; RE_allometric_, running economy allometrically scaled oxygen cost; RE_ec_, running economy energy cost; RE_ec-wb_, running economy expressed as energy cost excluding work of breathing.

None of the MotionMetrix biomechanical variables, such as cadence (1.2%), ground contact time (−2.3%), vertical displacement (−1%), overstride (−6.7%), or vertical force (2.6%), differed significantly between pre- and post-test for any variable (*p* > 0.05 for all the variables).

### Relationship between changes in RE and other variables

To examine whether differences in waiting time between the end of the race and the post-test influenced changes in RE, a Pearson’s correlation analysis between ΔRE_ox_ and waiting time was conducted. The results of this analysis showed that there was no significant correlation between ΔRE_ox_ and waiting time (r = 0.039; *p* = 0.898).

### Physiological and performance-related measurements

Participants’ pre-test measurements of VO_2_max (mL·min^−1^·kg^−1^; r = 0.181, *p* = 0.573), VO_2_ at θLT (r = 0.253, *p* = 0.428), and RER at 14 km h^−1^ (r = −0.178, *p* = 0.560) did not significantly relate to ΔRE_ox._ Furthermore, participants’ performance did not relate to ΔRE_ox_ (r = −0.440, *p* = 0.132). Moreover, no significant relationships between ΔRE_ox_ and ΔWB (r = 0.470, *p* = 0.105), ΔVE (r = 0.481, p = 0.096), Δbody mass (r = −0.493, *p* = 0.087), ΔH-la (r = 0.496, *p* = 0.084), and ΔRER (r = −0.388, *p* = 0.191) were observed.

### Biomechanical measurements

No significant relationships were found between changes in biomechanical variables obtained from MotionMetrix and ΔRE_ox_. ΔCadence (r = 0.215, *p* = 0.480), Δ ground contact time (r = 0.234, *p* = 0.442), ΔOverstride (r = −0.250, *p* = 0.411), Δvertical force (r = −0.174, *p* = 0.569), and Δvertical displacement (r = −0.286, *p* = 0.343) did not relate to ΔRE_ox_.

## Discussion

The main findings indicate that the optimal regression model (*R* ^2^ = 0.918) included fat% at 14 km h^−1^, VO_2_ at θLT, and RE_allometric_ as the strongest predictors of performance. The race increased VO_2_ relative to body mass by 3.4% and RE_ox_ by 3.4%. Post-race substrate utilization showed a 62.22% increase in fat oxidation and a 25.32% decrease in carbohydrate use, reducing RER by 6.18%. Biomechanical parameters did not change from pre- to post-race.

### Race performance

Consistent with the classical endurance performance model (Bassett and Howley, 2000), performance in the 30 km XC Lidingöloppet correlated with VO_2_max, fractional utilization (speed and VO_2_ at θLT), speed at RCP, and RE (allometrically scaled oxygen and energy cost), but not RE without scaling. Additionally, race performance was correlated to substrate utilization at submaximal speed and fat mass. However, a regression with only VO_2_max, RE, and fractional utilization was limited by multicollinearity (*R* ^2^ = 0.834), whereas a model including fat% at 14 km h^−1^, VO_2_ at θLT, and RE_allometric_ explained more variance (*R* ^2^ = 0.918).

Although VO_2_max correlated strongly with performance, it was excluded from the optimal regression, somewhat contradicting previous trail-running studies (Ehrström et al., 2018; Alvero-Cruz et al., 2019; Pastor et al., 2022). As described by Costill et al. (1973), VO_2_max is not a good predictor among athletes with similar VO_2_max; in our cohort, values were homogeneous, except for two outliers. Thus, VO_2_max did not add explanatory power, despite its correlation. Likewise, vVO_2_max—linked to VO_2_max and RE and shown to predict trail-running performance (Alvero-Cruz et al., 2019; Coates et al., 2021; Sabater-Pastor et al., 2023)—also correlated with performance but was excluded. Nonetheless, VO_2_max and vVO_2_max remain important determinants of performance.

RE_allometric_ and RE_ec_ correlated with performance and featured in our regression, contrasting earlier trail-running studies of long races (Ehrström et al., 2018; Alvero-Cruz et al., 2019; Pastor et al., 2022; Coates et al., 2021) and short races (Björklund et al., 2019). Those studies, except for that by Ehrström et al. (2018), who measured incline RE, assessed RE on level terrain despite greater elevation gain and technical demands, which may have obscured its impact. Longer races may prioritize fractional utilization and muscle preservation over RE (Millet et al., 2012; Pastor et al., 2022; Coates et al., 2021). Lidingöloppet’s moderate elevation and technicality resemble hilly road races, where RE strongly predicts marathon performance (Barnes and Kilding, 2015). Importantly, only allometrically scaled oxygen cost was related to performance, implying that VO_2_/body mass is not strictly proportional (Svedenhag, 1995) across a wide mass range (the body mass range in this investigation was 54–83.6 kg). Additionally, RE_ec_ correlated with performance, underscoring the role of substrate utilization in RE (Fletcher et al., 2009).

θLT and RCP were strongly related to performance; VO_2_ at θLT featured in the optimal model, aligning with the study by Scheer et al. (2019). Conversely, VT did not predict performance in other trail races (Ehrström et al., 2018; Alvero-Cruz et al., 2019; Pastor et al., 2022), likely due to greater technical difficulty, elevation gain, and longer race duration, where low intensity diminishes the influence of VT (Millet et al., 2012). In Lidingöloppet’s shorter, less severe course, participants ran at 93% ± 5% of vθLT, indicating race pace and performance VO_2_ near vθLT and VO_2_ at θLT.

Substrate utilization correlated with performance; higher fat and lower carbohydrate oxidation at submaximal speeds likely spare glycogen. Pastor et al. (2022) also confirmed the importance of substrate utilization.

Fat mass was positively related to performance, consistent with previous studies (Alvero-Cruz et al., 2019; Pastor et al., 2022; Björklund et al., 2019). Higher fat mass increases gravitational work without enhancing capacity, explaining this relationship.

Parameters from the Stryd device (power output, ground contact time, vertical oscillation, leg spring stiffness, cadence, and step length) did not correlate with performance and were excluded, simplifying practical assessments by relying solely on metabolic-cart data.

### Changes between pre- and post-race

RE_ox_ significantly increased post-race, while absolute oxygen cost, RE_allometric_, and RE_ec_ remained unchanged.

These changes reflect increased fat reliance: a 62.22% increase in fat utilization demands more oxygen per energy yield, increasing RE_ox_, while RE_ec_ remains stable. RE_allometric_ also remains constant due to non-linear body mass scaling. Post-race, H-la increased, RER decreased (reflecting altered substrate use), WB increased, and body mass decreased. VE and biomechanical parameters did not change, although biomechanical responses varied individually.

Absolute oxygen cost did not increase post-race; instead, reduced body mass elevated relative oxygen cost and RE_ox_. Allometric scaling neutralized this change, suggesting that body mass influences relative oxygen cost (Svedenhag, 1995), although ΔRE_ox_ was not significantly related to Δbody mass, contradicting this notion. Maintaining the same absolute oxygen uptake while carrying less body mass inevitably increases RE_ox_ (mL·kg^-1^·km^-1^), which can give the appearance of poorer running performance. At the same time, the substantially decreased RER implies greater fat utilization, a substrate that requires more oxygen per unit of energy produced than carbohydrate oxidation. This indicates that the post-race increase in RE_ox_ may reflect the combined influence of body weight loss (via sweat and glycogen depletion) and a substrate shift toward greater fat oxidation once glycogen availability is reduced. However, ΔRE_ox_ did not correlate with ΔRER. Other studies show large RER shifts with increased energetic cost (Kyröläinen et al., 2000; Nicol et al., 2007; Sabater Pastor et al., 2021), likely due to longer, more demanding races, although short trials also report notable oxygen cost increases (Candau et al., 1998). Variations in race elevation, duration, population, and test conditions likely explain discrepancies. The lack of significant VE change and absence of ΔRE_ox_ correlations with ΔVE or ΔWB are in contrast with prior findings linking ΔVE and ΔRE (Thomas et al., 1995). Moreover, unchanged biomechanics and their non-association with ΔRE_ox_ align with studies showing no relation between RE and biomechanical changes (Hunter and Smith, 2007; Kyröläinen et al., 2000; Nicol et al., 2007). Thus, RE_ox_ changes might be likely driven by body mass loss (sweating and glycogen depletion) and decreased RER, requiring more oxygen for fat oxidation.

The performance level did not correlate with ΔRE_ox_, consistent with findings of Sabater Pastor et al. (2021), indicating that performance does not influence the degree of RE degradation in long trail races or XC races such as Lidingöloppet. This contradicts hypotheses linking RE deterioration and “fatigue resistance” (Jones et al., 2021; Brueckner et al., 1991), warranting further studies. Aerobic fitness, measured as VO_2_max, θLT, and submaximal running substrate use, also did not influence ΔRE_ox_. Pre-test RE_ox_ negatively correlated with ΔRE_ox_: runners with higher initial oxygen cost changed the least, and only two with high pre-test RE_ox_ reduced cost post-race (Figure 2). Notably, the top performer had the best pre-race RE_ox_ but the largest worsening (+23%), suggesting that higher initial RE_ox_ reserves allow a greater margin for decline. This variability underscores the importance of individual responsiveness post-race. This challenges the principle of Jone and s (2024) that the best resilience (least decline in critical power/velocity) confers an advantage. Although we did not consider the increase in energy cost at the individual critical speed or changes in critical power/velocity, our findings suggest that the subject with the best RE_ox_ in a fresh state might still have an advantage despite the post-competition decline. However, the heterogeneity of our sample makes it difficult to draw definitive conclusions, and further investigation is warranted.

### Limitations

There are some methodological limitations in the present study that need to be considered and discussed. The only speed tested that was common to all runners included in the study was 14 kmh^-1^; therefore, it was not possible to capture physiological and biomechanical responses post-race at different intensities. During the post-tests, waiting time before testing was minimized, resulting in a practical upper limit on the number of participants. On average, post-race measurements were delayed by 45:15 ± 24:28 (min:sec). This relatively long and inconsistent waiting period may have allowed participants to recover to varying degrees, potentially affecting the recorded physiological values and masking the true magnitude of immediate fatigue or metabolic disturbances. To avoid waiting times exceeding ∼45 min, approximately 15 participants could have been included, depending on the ranges in finishing times. With a sample size of 13, however, the statistical power to detect moderate effect sizes (Cohen’s d = 0.6) is low (0.512), and large effect sizes (Cohen’s d = 0.8) are required for sufficient power (>0.8). This suggests a likely large risk of type 2 errors in this study, indicating that moderate effect sizes do not reach significance due to the sample size. Accordingly, observed non-significant changes with moderate effect sizes should be interpreted with caution before conclusions are drawn. However, previous studies (Candau et al., 1998; Hunter and Smith, 2007; Kyröläinen et al., 2000; Nicol et al., 2007; Sabater Pastor et al., 2021; Lazzer et al., 2015) investigating the effect of fatigue caused by running on RE had sample sizes of 7–26. Another limitation in the sample size was that the group included one elite runner who was an outlier. When this elite runner is excluded, the regression model’s explained variance decreases from 91.8% to 84.9%, indicating that the presence of this outlier has an impact on our analyses. The group otherwise included runners ranging from amateur to well-trained, which limits the conclusions that can be drawn regarding the effects of fatigue in elite runners and the differences in responses between elite and well-trained athletes. However, the observed relationships did not differ much from previous research, and the participants’ results were logical and therefore included. It is also likely that adding more data points from a broader range of athletes would yield similar results. In our sample, there was only one female athlete; therefore, the results may not be representative of the female population, although the exclusion of this subject did not alter the variance explained by the model, which remained at 91.8%. Finally, the lack of a control group restricts the ability to establish causal relationships. Future studies should include larger sample sizes to increase statistical power, address sex differences, and explore the potential deterioration of running economy at different running speeds.

## Conclusion

This study showed that performance in the 30 km XC-running race Lidingöloppet was related to RE, VO_2_max, vVO_2_max, θLT, along with RCP, substrate utilization, and fat mass. The best performance model could explain 91.8% of the variance in performance and included VO_2_ at θLT, fat% at 14 km h^−1^, and RE_allometric_. Physiological variables included in the classical model of performance were also related to performance in XC running; however, exchanging VO_2_max with fat% at 14 km h^−1^ increased the model’s ability to explain variance in performance. This could practically be used by athletes and coaches to better direct training to improve performance in this type of XC race. The oxygen cost of running, RE_ox_, increased post-race, likely due to decreased body mass and increased fat oxidation. No biomechanical changes were observed post-race, and biomechanical changes could not explain increased RE_ox_. The participants’ aerobic fitness and performance level did not influence or correlate with the degree of change in RE_ox_.

Larger and more diverse studies are needed before generalizing practical applications. The present results, while important, should be considered exploratory due to methodological constraints such as the limited sample size and variability in post-race measurement timing. However, based on this study, it can be suggested that practitioners prioritize exercise and nutritional strategies that enhance fat oxidation at submaximal intensities and incorporate both plyometric and resistance training to improve allometrically scaled running economy—thereby elevating speed at the lactate threshold. Furthermore, since VO_2_ at θLT, fat% at 14 km h^−1^, and RE_allometric_ emerged as the strongest performance predictors, we recommend relying on metabolic-cart measurements rather than wearable-derived biomechanical metrics for XC performance assessment and training guidance. Future research is still needed to explain the observed changes in RE_ox_ and why they differ inter-individually. Moreover, further research is required to determine whether the performance level is related to the deterioration in RE.

## Data Availability

The original contributions presented in the study are publicly available. This data can be found here: https://doi.org/10.6084/m9.figshare.29313851.v1.

## Abbreviations

H-la, Blood Lactate Concentration (mmol·L^-1^); PETCO_2_, End-Tidal Partial Pressure of Carbon Dioxide; PETO_2_, End-Tidal Partial Pressure of Oxygen; RCP, Respiratory Compensation Point; RER, Respiratory Exchange Ratio; RE, Running Economy; RE_allometric_, Allometrically Scaled Running Economy expressed in mL·kg^0.75^·km^-1^; REec, Running Economy (energy cost) expressed in kcal·kg^-1^·km^-1^; REec-wb, Running Economy expressed as Energy Cost excluding Work of Breathing (kcal·kg^-1^·km^-1^); REox, Running Economy expressed as Oxygen Cost in mL·kg^-1^·km^-1^; VE, Ventilation; vRCP, Velocity at Respiratory Compensation Point; vVO_2_max, Velocity at VO_2_max; vθLT, Velocity at Estimated Lactate Threshold; wb, Work of Breathing (energy cost, kcal·kg^-1^·km^-1^); θLT, Estimated Lactate Threshold.
